# Experimental Efficacy of a Novel Combined Vaccine of Porcine Circovirus Types 2a/d, *Mycoplasma hyopneumoniae* and *M. hyorhinis*

**DOI:** 10.3390/vaccines13090951

**Published:** 2025-09-05

**Authors:** Jeongmin Suh, Sehyeong Ham, Hyejin Na, Youngkook You, Bumsoo Park, Chanhee Chae

**Affiliations:** 1 Department of Veterinary Pathology, College of Veterinary Medicine, Seoul National University, Gwanak-ro 1, Gwanak-gu, Seoul 08826, Republic of Korea; wayne21818@gmail.com (J.S.); hsh6926@snu.ac.kr (S.H.); skwls1212@snu.ac.kr (H.N.); 2 CARESIDE Co., Ltd., Seongnam-si 13209, Republic of Korea; careside@careside.co.kr (Y.Y.); pbs1776@gmail.com (B.P.)

**Keywords:** enzootic pneumonia, *Mycoplasma hyopneumoniae*, *Mycoplasma hyorhinis*, porcine circovirus type 2a, porcine circovirus type 2d, porcine respiratory disease complex, vaccine

## Abstract

Background: The efficacy of a novel combined vaccine targeting porcine circovirus types 2a/d (PCV2a/d), *Mycoplasma hyopneumoniae*, and *M. hyorhinis* was evaluated in a controlled challenge study. Methods: A total of 45 pigs were randomly allocated into nine groups (five pigs per group). Vaccinated groups received a single 2 mL intramuscular dose of the combined vaccine and were subsequently challenged with PCV2a, PCV2d, *M. hyopneumoniae*, and *M. hyorhinis*. Unvaccinated groups received a single 2 mL intramuscular dose of phosphate-buffered saline (0.01 M, pH 7.4). Growth performance, systemic adaptive immune (humoral and cellular) responses, viremia, laryngeal and nasal mycoplasma loads, and histopathological lesions were assessed. Results: Vaccinated pigs exhibited enhanced growth performance and elicited systemic immune responses, including both humoral and cellular immunity, against all four pathogens. Vaccination also significantly reduced viremia, mycoplasmal loads in laryngeal and nasal swabs, and the severity of associated lesions compared with unvaccinated controls. Conclusions: These results indicated that the combined vaccine was efficacious and conferred protection against PCV2a, PCV2d, *M. hyopneumoniae*, and *M. hyorhinis* challenge under experimental conditions. This combined vaccine represented an effective strategy to enhance growth performance and control complex co-infection in swine populations.

## 1. Introduction

Porcine circovirus type 2 (PCV2) is the smallest circular DNA virus identified to date, yet it remains an important pathogen associated with multiple syndromes and diseases collectively referred to as porcine circovirus-associated diseases (PCVAD) [1,2,3]. PCVAD continues to cause significant economic losses to the global pig industry. Currently, eight distinct genotypes of PCV2 have been proposed (PCV2a to PCV2h) [4]. Of these, PCV2a, PCV2b, and PCV2d are the most prevalent, with PCV2d predominating in most of swine-producing countries [4].

*Mycoplasma hyopneumoniae* is the primary etiological agent of enzootic pneumonia and a key contributor to the porcine respiratory disease complex (PRDC), an economically important condition in pigs worldwide [5]. *M. hyorhinis* is recognized as a major cause of polyserositis and arthritis [5]. More recently, *M. hyorhinis* has emerged an important concern in swine production due to its association with increased morbidity and mortality in the postweaning period (personal observation, Chae).

Vaccination with a combination product targeting PCV2 and *M. hyopneumoniae* combination product is widely practiced worldwide to control the two pathogens, particularly in Asian pig-rearing countries. In general, PCV2 vaccines provide better protection against homologous genotypes than against heterologous challenge strains [6,7,8,9], accumulating evidence suggest that they are “leaky vaccines” [10,11]. Most commercially available PCV2 vaccines are still based on PCV2a or PCV2b, whereas PCV2d has become the predominant circulating field genotype in many Asian countries, including China, Korea, Taiwan, Thailand, and Vietnam [12,13,14,15,16]. Consequently, there is a need for commercial vaccines incorporating PCV2d to achieve more effective control under current field conditions.

Recently, a novel commercial combined vaccine containing PCV2a, PCV2d, *M. hyopneumoniae*, and *M. hyorhinis* was introduced to the Korean market, where PCV2d predominates and cases of polyserositis caused by *M. hyorhinis* is increasing. The objective of the present study was to evaluate the efficacy of this novel combined vaccine in conferring protection against experimental challenge with PCV2a, PCV2d, *M. hyopneumoniae*, and *M. hyorhinis*.

## 2. Materials and Methods

### 2.1. Animals

Forty-five clinically healthy piglets, aged 18 days, were obtained from a commercial sow farm. The source herd had been confirmed free of *Mycoplasma hyopneumoniae* and porcine reproductive and respiratory syndrome virus (PRRSV) through routine serological monitoring, supported by long-term clinical observation and slaughter checks. At the time of arrival, all piglets underwent serological screening and tested negative for PCV2 (Porcine Circovirus type 2 Antibody Test, BioChek B.V., Reeuwijk, The Netherlands), PRRSV (HerdChek PRRS X3 Ab test, IDEXX Laboratories Inc., Westbrook, ME, USA), *Mycoplasma hyopneumoniae* (ID Screen^®^ *Mycoplasma hyopneumoniae* Competition ELISA, ID.Vet, Grabels, France), and *M. hyorhinis* (in-house ELISA). In addition, real-time polymerase chain reaction (PCR) assays confirmed the absence of PCV2 and PRRSV viremia, as well as *M. hyopneumoniae* and *M. hyorhinis* in the pharyngeal samples.

### 2.2. Vaccine

A combined vaccine, XENOVAX PCM4 (CARESIDE CO., LTD., Seongnam-si, Gyeonggi-do, Republic of Korea, http://www.careside.co.kr), consists of four recombinant proteins, PCV2d, PCV2a, T-BLS (*Brucella lumazine* synthase + T-cell; ≥RP 1.0), and *M. hyopneumoniae* p65 protein-together with two inactivated whole-cell bacterin, *M. hyopneumoniae* (≥RP 1.0) and *M. hyorhinis* (≥RP 1.0). The PCV2a and PCV2d capsid proteins were expressed in an *Escherichia coli* system and self-assembled into virus-like particles that are morphologically indistinguishable from native PCV2. The vaccine was adjuvanted with 12% of Emulcigen_BCL (MVP adjuvants, Omaha, NE, USA) and Quil-A (100 μg/mL, InvivoGen, Hong Kong, China). XENOVAX PCM4 is administered intramuscularly as a single 2.0 mL dose from three weeks of age onward.

### 2.3. Experimental Design

All animal procedures were reviewed and approved by the Seoul National University Institutional Animal Care and Use, and Ethics Committee (SNU-211222-3-1). The challenge study was conducted incompliance with the Republic of Korea’s Animal, Plant & Fisheries Quarantine & Inspection Agency (QIA, http://www.qia.go.kr) registration guidelines. A total of 45 pigs were randomly allocated into nine experimental groups (five pigs per group; two males and three females) (Table 1). Each group was kept in a separate isolation room under uniform housing conditions, with unrestricted access to feed and water.

At −21 days post challenge (dpc, 21 days of age), pigs in the Vac/ChMhp, Vac/Ch2dMhr, Vac/Ch2a, and Vac/Ch2d groups were intramuscularly immunized with 2.0 mL of the combined vaccine (Xenovax PCM4, Careside, Seongnam-si, Republic of Korea). Pigs in the UnVac/ChMhp, UnVac/Ch2dMhr, UnVac/Ch2a, UnVac/Ch2d, and UnVac/UnCh groups received an intramuscular injection of 2.0 mL of phosphate-buffered saline (PBS, 0.01 M, pH 7.4).

At 0 days post challenge (42 days of age), pigs in the Vac/ChMhp and UnVac/ChMhp groups were challenged with *M. hyopneumoniae* (strain SNU98703). Prior to inoculation, pigs were anesthetized intramuscularly with a combination of 2.2 mg/kg xylazine hydrochloride (Rompun, Bayer Healthcare, Shawnee Mission, KS, USA), 2.2 mg/kg tiletamine hydrochloride, and 2.2 mg/kg zolazepam hydrochloride (Zoletil 50, Virbac, Carros, France). The challenge was performed intratracheally with 7 mL of *M. hyopneumoniae* (strain SNU98703) culture medium containing 10^7^ color changing units/mL, following the procedure described previously [17].

Pigs in the Vac/Ch2dMhr and UnVac/Ch2dMhr groups were first challenged with PCV2d (SNUVR202003 strain, GenBank no. MZ440695, 5th passage in PCV-free PK-15 cell lines), followed five hours later by *M. hyorhinis* (strain CS401-001). For the PCV2d challenge, pigs received 3 mL of inoculum intranasally containing 1.2 × 10^5^ TCID_50_/mL (50% tissue culture infective dose/mL). Five hours post-inoculation, pigs were anesthetized intramuscularly as described above and subsequently challenged with *M. hyorhinis* (strain CS401-001) by intratracheal (2 mL) and intraperitoneal (2 mL) inoculation of culture medium containing 10^9^ color changing units/mL, following previously established protocols [18].

Pigs in the Vac/Ch2a and UnVac/Ch2a groups were challenged intranasally with a 3 mL of inoculum containing 1.2 × 10^5^ TCID_50_/mL of PCV2a (SNUVR100032 strain, GenBank no. KF871067, 5th passage in PCV-free PK-15 cell lines). Similarly, pigs in the Vac/Ch2d and UnVac/Ch2d groups were challenged intranasally with a 3 mL of inoculum containing 1.2 × 10^5^ TCID_50_/mL of PCV2d (SNUVR202003 strain, GenBank no. MZ440695, 5th passage in PCV-free PK-15 cell lines).

At 21 days post challenge (63 days of age), all pigs were sedated with intravenous sodium pentobarbital and subsequently euthanized by electrocution as previously described [19]. During necropsy, tissue samples from each pig were collected, fixed in 10% neutral buffered formalin, and processed for paraffin embedding.

### 2.4. Collection of Samples

Sampling involved the collection of blood, nasal swabs, and laryngeal swabs from all animals at −21, 0, 7, 14, and 21 days post challenge.

### 2.5. Clinical Scoring

Pigs were monitored daily for clinical signs, and severity scores were recorded weekly using a defined scale: 0 (normal), 1 (rough haircoat), 2 (rough haircoat and dyspnea), 4 (severe dyspnea and abdominal breathing), and 6 (death). Scoring was performed by examiners blinded to treatment groups.

### 2.6. Growth Performance

Body weight was recorded for each pig at three time points: −21 (corresponding to 21 days of age), 0 (42 days of age), and 21 (63 days of age) days post challenge. Average daily weight gain (ADWG; g/pig/day) was calculated for three intervals: (i) from 21 to 42 days of age, (ii) from 42 to 63 days of age, and (iii) from 21 to 63 days of age. For each interval, ADWG was derived as (final weight − initial weight)/number of days.

### 2.7. PCV2 DNA Quantification in Serum Samples

Serum DNA was isolated using the QIAamp DNA Mini Kit (QIAGEN, Valencia, CA, USA) according to the manufacturer’s instructions. Real-time PCR was used to quantify genomic copy numbers of PCV2a and PCV2d [20,21].

### 2.8. M. hyopneumoniae DNA Quantification in Laryngeal Samples

DNA was extracted from laryngeal samples using the QIAamp DNA Mini Kit (QIAGEN, Valencia, CA, USA) according to the manufacturer’s instructions. Real-time PCR was used to quantify genomic copy numbers of *M. hyopneumoniae* [22].

### 2.9. M. hyorhinis DNA Quantification in Nasal Samples

DNA was extracted from nasal samples using the QIAamp DNA Mini Kit (QIAGEN, Valencia, CA, USA) according to the manufacturer’s instruction. Real-time PCR was used to quantify genomic copy numbers of *M. hyorhinis* [23].

### 2.10. PCV2 Serology

Serum antibody responses to PCV2 were evaluated using a commercial enzyme-linked immunosorbent assay (ELISA; BioChek B.V., Reeuwijk, The Netherlands). According to the manufacturer’s criteria, samples with the sample-to-positive (S/P) ratio of ≥0.5 were considered positive. In addition, serum viral neutralization tests were performed in 96-well microtitration plates using PK-15 cells as the indicator cells [24,25].

### 2.11. M. hyopneumoniae Serology

Antibody responses to *Mycoplasma hyopneumoniae* were evaluated in serum samples using a commercially available competitive ELISA kit (ID Screen^®^ *Mycoplasma hyopneumoniae* Competition ELISA, ID.Vet, Grabels, France) following the manufacturer’s protocol. Samples with the sample-to-negative (S/N) ratio ≤ 0.5 were considered positive.

### 2.12. M. hyorhinis Serology

An in-house ELISA was performed to detect *Mycoplasma hyorhinis*-specific antibodies in serum. Purified p70 protein was diluted in carbonate-bicarbonate buffer and coated onto 96-well MaxiSorp plates (Nunc, Invitrogen, Carlsbad, CA, USA) at 0.1 ng per well, followed by overnight incubation at 4 °C. Plates were then washed three times with PBST (0.05% Tween 20 in PBS) between incubation steps. Blocking was performed with 50 µL/well of 2% skim milk in PBST for 1 h at 37 °C. Serum samples and controls, diluted 1:100 in PBST, were added in duplicate (50 µL per well) and incubated for 1 h at 37 °C. Goat anti-swine IgG conjugated to peroxidase (KPL, Seracare Life Sciences, Milford, MA, USA) was added at a 1:2000 dilution in 0.2% skim milk and incubated for 1 h at 37 °C. After washing, plates were developed with 50 µL/well of SureBlue TMB substrate (Seracare) for 10 min at room temperature, followed by 50 µL/well stop solution (Seracare). Absorbance at 450 nm was measured within 15 min using a microplate reader. Samples with a sample-to-positive (S/P) ratio ≥ 0.3 were classified as positive.

### 2.13. Quantification of Antigen-Specific IFN-γ-Secreting Cells

An enzyme-linked immunospot (ELISpot) assay was performed to quantify interferon-γ secreting cells (IFN-γ-SC) specific to PCV2a, PCV2d, *Mycoplasma hyopneumoniae*, and *M. hyorhinis*. Peripheral blood mononuclear cells (PBMC) were stimulated with the respective challenge strains. [21,26]. Results were expressed as the number of IFN-γ-SC per million PBMC.

### 2.14. Pathology

For gross lesion scoring, two pathologists at the Seoul National University, blinded to treatment groups, evaluated the extent of lung involvement and calculated the percentage of the lung affected by pneumonia [27]. Lesions of the hepatic serosa were assessed separately based on the percentage of affected parts (0 = none; 1 = 0–30%; 2 = 31–60%; 3 = 61–100%), following previously described methods [18].

For microscopic evaluation, lung, lymphoid, and hepatic tissue sections were evaluated independently by two blinded veterinary pathologists. Lung lesions were scored based on the presence of peribronchial and peribronchiolar lymphoid hyperplasia, and degree of inflammation in the lamina propria of bronchi and bronchioles, using a scale of 0 to 6 (0 = normal; 1 = mild multifocal; 2 = mild diffuse; 3 = moderate multifocal; 4 = moderate diffuse; 5 = severe multifocal; 6 = severe diffuse) [28]. Real-time PCR was performed to confirm mycoplasmal pneumonia as previously described [22]. Lymphoid lesions were scored based on the presence of lymphoid depletion and inflammation, using a scale of 0 to 5 (0 = normal; 1 = mild lymphoid depletion; 2 = mild to moderate lymphoid depletion and histiocytic replacement; 3 = moderate diffuse lymphoid depletion and histiocytic replacement; 4 = moderate to severe lymphoid depletion and histiocytic replacement; 5 = severe lymphoid depletion and histiocytic replacement) [29]. Hepatic lesions were assessed for fibrinous perihepatitis using a scoring scale of 0 to 6 (0 = normal; 1 = mild multifocal; 2 = mild diffuse; 3 = moderate multifocal; 4 = moderate diffuse; 5 = severe multifocal; 6 = severe diffuse).

### 2.15. Statistical Analysis

Real-time PCR results and neutralizing antibody titers were log-transformed to base 10 and base 2, respectively, before analysis. Normality was assessed using the Shapiro–Wilk test. If the data were normally distributed, one-way ANOVA was performed to compare groups at each time point, followed by Tukey’s post hoc test. For non-normally distributed data, the Kruskal–Wallis test was applied, and statistically significant results were further analyzed using the Mann–Whitney U test. A *p*-value of less than 0.05 was considered statistically significant.

## 3. Results

### 3.1. Decreased Respiratory Clinical Signs

Within the *M. hyopneumoniae* challenged groups, pigs in the Vac/ChMhp group had significantly decreased the respiratory clinical scores (*p* < 0.05) at 14 and 21 days post challenge compared with pigs in the UnVac/ChMhp group. Within the PCV2a challenged groups, pigs in the Vac/Ch2a experienced a significant decrease in respiratory clinical scores (*p* < 0.05) at 14 and 21 days post challenge compared with pigs in the UnVac/Ch2a group. Within the PCV2d challenged groups, pigs in the Vac/Ch2d had a significant decrease in respiratory clinical scores (*p* < 0.05) at 14 and 21 days post challenge compared with pigs in the UnVac/Ch2d group. Within the PCV2d and *M. hyorhinis* challenged groups, pigs in the Vac/Ch2dMhr had a significant decrease in respiratory clinical scores (*p* < 0.05) at 14 and 21 days post challenge compared with pigs in the UnVac/Ch2dMhr group.

### 3.2. Improved Growth Performance

The body weight of the pigs did not differ significantly among 9 groups at −21 days post challenge (21 days of age; the time of vaccination) and 0 (42 days of age; the time of challenge) days post challenge. Within the *M. hyopneumoniae* challenged pigs, Vac/ChMhp treatment resulted in significant (*p* < 0.05) improvement in body weight at 21 days post challenge (63 days of age) compared with pigs in the UnVac/ChMhp group. Pigs in the Vac/ChMhp group had a significant improvement in their ADWG (*p* < 0.05) from 0 (42 days of age) to 21 (63 days of age) days post challenge and from −21 (21 days of age) to 21 (63 days of age) days post challenge compared with pigs in the UnVac/ChMhp group. Within the PCV2d and *M. hyorhinis* challenged group, pigs in the Vac/Ch2dMhr had a significant improvement in body weight (*p* < 0.05) at 21 days post challenge (63 days of age) compared with pigs in the UnVac/Ch2dMhr group. Pigs in the Vac/Ch2dMhr group had a significant improvement in ADWG (*p* < 0.05) from 0 (42 days of age) to 21 (63 days of age) days post challenge and from −21 (21 days of age) to 21 (63 days of age) days post challenge compared with pigs in the UnVac/Ch2dMhr group (Table 2).

### 3.3. PCV2 DNA Genomic Copy Numbers in Serum Samples

**Figure 1:**
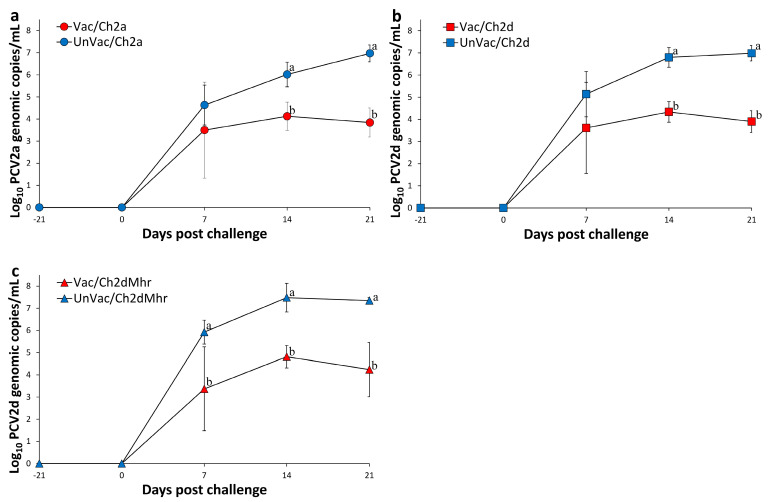
Mean genomic copy numbers of PCV2 DNA in serum samples across experimental groups. (**a**) PCV2a loads in Vac/Ch2a vs. UnVac/Ch2a groups. (**b**) PCV2d loads in Vac/Ch2d vs. UnVac/Ch2d groups. (**c**) PCV2d loads in Vac/Ch2dMhr vs. UnVac/Ch2dMhr groups. Variation is expressed as the standard deviation. Different superscripts (a and b) indicate significant differences (*p* < 0.05) between groups within each pair.

In pigs challenged with PCV2a, no PCV2a DNA was detected in serum at either the time of vaccination or viral challenge. A significant reduction (*p* < 0.05) in serum PCV2a genomic copies was observed in the vaccinated and challenged group (Vac/Ch2a) at 14 and 21 days post challenge compared to the unvaccinated and challenged group (UnVac/Ch2a) (Figure 1a). Similarly, in the PCV2d challenge group, serum samples were negative for PCV2d DNA prior to both vaccination and challenge. Vaccinated pigs (Vac/Ch2d) exhibited significantly (*p* < 0.05) lower PCV2d DNA levels at 14 and 21 days post challenge relative to the UnVac/Ch2d group (Figure 1b). Within the PCV2d and *M. hyorhinis*-challenged pigs, no PCV2d DNA was present in serum prior to treatment. The experiment resulted in a significant (*p* < 0.05) reduction in the amount of PCV2d DNA measured from serum of the vaccinated-challenged groups (Vac/Ch2dMhr) at 7, 14, and 21 days post challenge when compared with their unvaccinated counterparts (UnVac/Ch2dMhr) (Figure 1c). Throughout the study, serum samples from the negative control group (UnVac/UnCh) remained free of detectable PCV2a and PCV2d DNA.

### 3.4. M. hyopneumoniae DNA Copy Numbers in Laryngeal Samples

**Figure 2:**
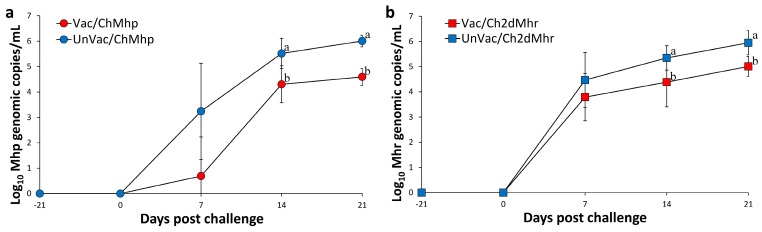
Mean genomic copy numbers of Mycoplasma DNA in challenged pigs. (**a**) *M. hyopneumoniae* (Mhp) loads in laryngeal samples of Vac/ChMhp vs. UnVac/ChMhp groups. (**b**) *M. hyorhinis* (Mhr) loads in nasal samples of Vac/Ch2dMhr vs. UnVac/Ch2dMhr groups. Variation is expressed as the standard deviation. Different superscripts (a and b) indicate significant differences (*p* < 0.05) between the two groups in each comparison.

At the time of vaccination and challenge, no *M. hyopneumoniae* DNA was detected in laryngeal swabs from any pigs. The experiment resulted in a significant (*p* < 0.05) reduction in the amount of laryngeal *M. hyopneumoniae* DNA from the vaccinated-challenged group (Vac/ChMhp) at 14 and 21 days post challenge compared to those in the unvaccinated and challenged group (UnVac/ChMhp). Throughout the experiment, no *M. hyopneumoniae* DNA was identified in laryngeal samples from the negative control group (UnVac/UnCh) (Figure 2a).

### 3.5. M. hyorhinis DNA Copy Numbers in Nasal Samples

At the time of vaccination and challenge, *M. hyorhinis* DNA was undetectable in nasal swabs from all pigs. The experiment resulted in a significant (*p* < 0.05) reduction in the amount of nasal *M. hyorhinis* DNA of vaccinated-challenged groups (Vac/ChMhr) at 14 and 21 days post challenge compared to the unvaccinated and challenged group (UnVac/ChMhr). No *M. hyorhinis* DNA was detected in nasal samples from the negative control group (UnVac/UnCh) throughout the study (Figure 2b).

### 3.6. Host Immune Responses Against PCV2

**Figure 3:**
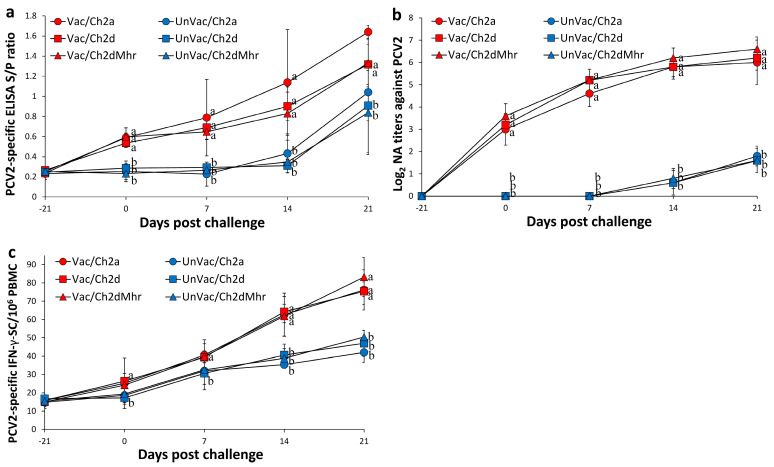
Immune responses against PCV2 in vaccinated and unvaccinated pigs following challenge. (**a**) Serum PCV2-specific antibody levels measured by ELISA in Vac/Ch2a, Vac/Ch2d, Vac/Ch2dMhr, UnVac/Ch2a, UnVac/Ch2d, and UnVac/Ch2dMhr groups. (**b**) Serum neutralizing antibody (NA) titers against PCV2 in the same groups. (**c**) Numbers of interferon-γ-secreting cells (IFN-γ-SC)/10^6^ PBMCs in response to PCV2 antigen stimulation across the six groups. Variation is expressed as the standard deviation. Different superscripts (a and b) indicate significant differences (*p* < 0.05) between groups.

From 0 to 21 days post-challenge, the vaccinated and challenged groups (Vac/Ch2a, Vac/Ch2d, and Vac/Ch2dMhr) exhibited significantly higher (*p* < 0.05) PCV2 ELISA S/P ratios compared to their unvaccinated counterparts (UnVac/Ch2a, UnVac/Ch2d, and UnVac/Ch2dMhr) (Figure 3a). PCV2a-specific neutralizing antibody (NA) titers in the vaccinated-challenged groups (Vac/Ch2a) were markedly elevated (*p* < 0.05) compared to the UnVac/Ch2a group from 0 to 21 days post challenge (Figure 3b). PCV2d-specific NA titers from the vaccinated-challenged groups (Vac/Ch2d and Vac/Ch2dMhr) were significantly higher (*p* < 0.05) relative to the UnVac/Ch2d and UnVac/Ch2dMhr groups from 0 to 21 days post challenge (Figure 3b).

PCV2a-specific IFN-γ-secreting cell (IFN-γ-SC) levels were significantly increased (*p* < 0.05) in the Vac/Ch2a group from 14 to 21 days post challenge compared to the UnVac/Ch2a group. PCV2d-specific IFN-γ-SC responses in the Vac/Ch2d group were also significantly elevated (*p* < 0.05) from 0 to 21 days post challenge relative to the UnVac/Ch2d group. PCV2d-specific IFN-γ-SC responses Vac/Ch2dMhr group were significantly elevated (*p* < 0.05) compared to the UnVac/Ch2dMhr group from 14 to 21 days post challenge. Throughout the study, the negative control group (UnVac/UnCh) showed only baseline PCV2 ELISA S/P ratios and IFN-γ-SC counts, and no detectable PCV2d-specific NA titers (Figure 3c).

### 3.7. Host Immune Responses Against M. hyopneumoniae

**Figure 4:**
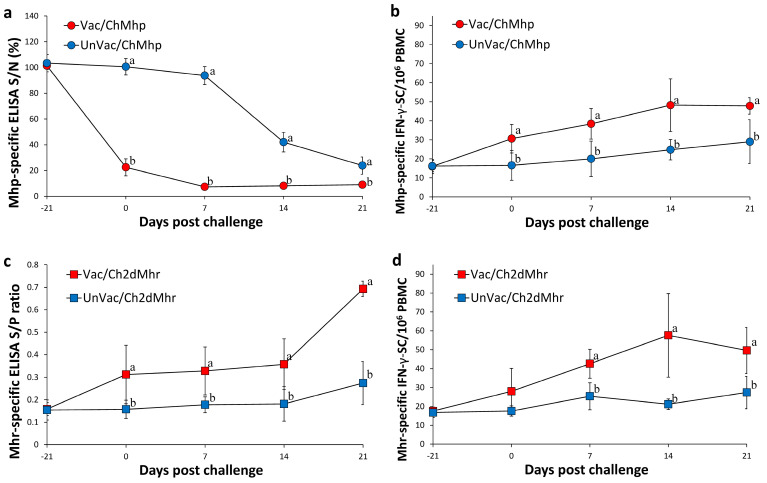
Immune responses against *Mycoplasma hyopneumoniae* (Mhp) and *M. hyorhinis* (Mhr) in vaccinated and unvaccinated pigs following challenge. (**a**) Serum antibody levels against Mhp in Vac/ChMhp vs. UnVac/ChMhp groups. (**b**) IFN-γ-secreting cells (IFN-γ-SC)/10^6^ PBMCs in response to Mhp stimulation in Vac/ChMhp vs. UnVac/ChMhp groups. (**c**) Serum antibody levels against Mhr in Vac/Ch2dMhr vs. UnVac/Ch2dMhr groups. (**d**) IFN-γ-secreting cells (IFN-γ-SC)/10^6^ PBMCs in response to Mhr stimulation in Vac/Ch2dMhr vs. UnVac/Ch2dMhr groups. Variation is expressed as the standard deviation. Different superscripts (a and b) indicate significant differences (*p* < 0.05) between groups in each comparison.

The vaccinated-challenged group (Vac/ChMhp) showed significantly reduced S/N ratios (*p* < 0.05) in the *M. hyopneumoniae*-specific ELISA compared to their unvaccinated-challenged counterparts (UnVac/ChMhp) from 0 to 21 days post challenge (Figure 4a). IFN-γ-secreting cell (IFN-γ-SC) counts specific to *M. hyopneumoniae* in the Vac/ChMhp group were markedly higher (*p* < 0.05) compared to the UnVac/ChMhp group from 0 and 21 days post challenge (Figure 4b). Both *M. hyopneumoniae*-specific S/N ratios and IFN-γ-SC levels remained at baseline in the negative control group (UnVac/UnCh) throughout the study.

### 3.8. Host Immune Responses Against M. hyorhinis

Pigs in the vaccinated-challenged group (Vac/Ch2dMhr) exhibited significantly elevated S/P ratios (*p* < 0.05) in the *M. hyorhinis*-specific ELISA compared to the unvaccinated group (UnVac/Ch2dMhr) from 0 to 21 days post challenge (Figure 4c). IFN-γ-SC responses to *M. hyorhinis* in Vac/Ch2dMhr were also significantly increased (*p* < 0.05) than UnVac/Ch2dMhr counterparts from 7 and 21 dpc (Figure 4d). As with other markers, baseline values of *M. hyorhinis*-specific ELISA S/P ratios and IFN-γ-SC responses were observed in the negative control animals (UnVac/UnCh) across all time points.

### 3.9. Reduced Pathological Lesion Severity

**Figure 5:**
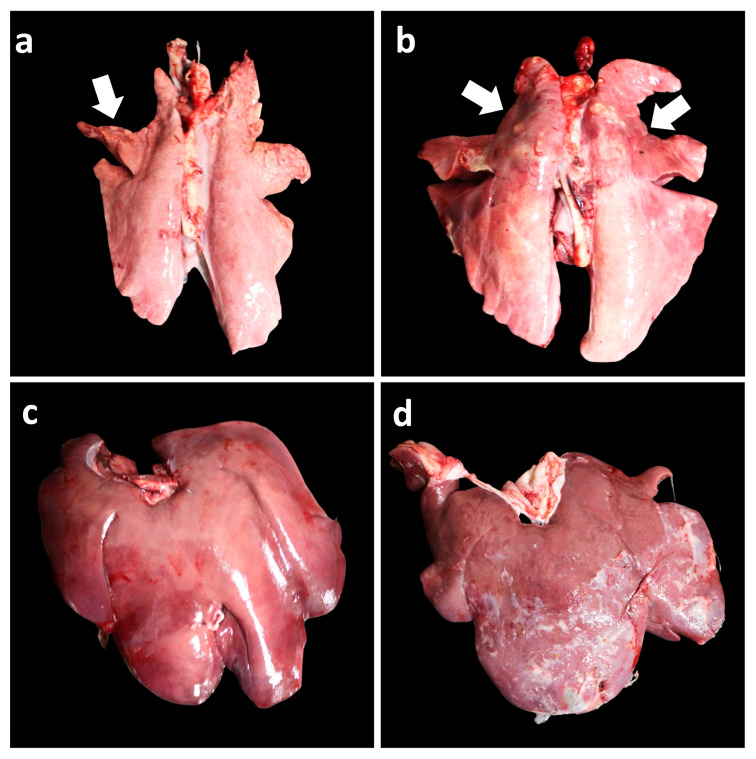
Gross pathological findings in vaccinated and unvaccinated pigs following challenge. (**a**) Minimal, well-demarcated, dark-red to purple firm parenchyma (arrow) in the lungs of the Vac/ChMhp group. (**b**) Severe, well-demarcated, dark-red to purple firm parenchyma (arrows) in the lungs of the UnVac/ChMhp group. (**c**) Minimal fibrinous exudate on the liver surface in the Vac/Ch2dMhr group. (**d**) Severe fibrinous exudate on the liver surface in the UnVac/Ch2dMhr group.

**Figure 6:**
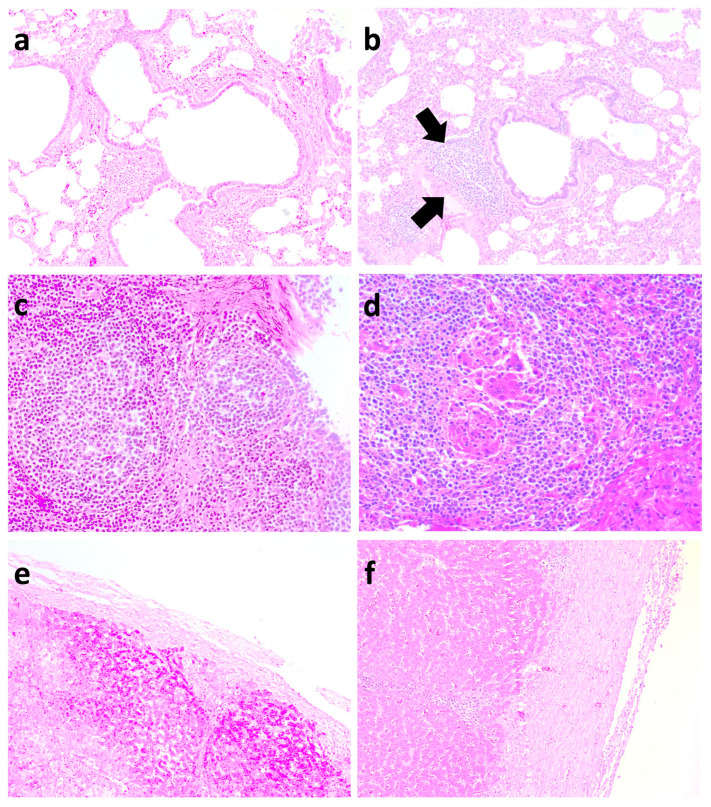
Histopathological lesions in vaccinated and unvaccinated pigs following challenge. (**a**) Minimal peribronchiolar lymphoid hyperplasia in the lungs of the Vac/ChMhp group. (**b**) Severe peribronchiolar lymphoid hyperplasia (arrows) in the lungs of the UnVac/ChMhp group. (**c**) Minimal lymphoid depletion in the lymphoid tissues of the Vac/Ch2dMhr group. (**d**) Severe diffuse lymphoid depletion with histiocytic replacement in the lymphoid tissues of the UnVac/Ch2dMhr group. (**e**) Minimal fibrinous perihepatic lesion in the Vac/Ch2dMhr group. (**f**) Severe fibrinous perihepatic lesion in the UnVac/Ch2dMhr group.

At 21 dpc, pigs in both the Vac/ChMhp and UnVac/ChMhp groups exhibited gross lung lesions, predominantly consisting of sharply demarcated, firm, dark-red to purple areas of consolidation (Figure 5a,b). Histologically, these lesions were characterized by marked peribronchiolar lymphoid hyperplasia (Figure 6a,b) in pigs in the Vac/ChMhp and UnVac/ChMhp groups. However, the extent of both macroscopic and microscopic pulmonary lesions was significantly diminished (*p* < 0.05) in the Vac/ChMhp group compared to its unvaccinated counterparts (UnVac/ChMhp) at 21 dpc (Table 3).

In pigs challenged with PCV2d and *M. hyorhinis,* minimal lymphoid depletion was observed in the Vac/Ch2dMhr group (Figure 6c), whereas severe diffuse lymphoid depletion with histiocytic infiltration was prominent in the UnVac/Ch2dMhr group (Figure 6d). Overall, vaccinated and challenged groups (Vac/Ch2a, Vac/Ch2d, and Vac/Ch2dMhr) exhibited significantly reduced (*p* < 0.05) lymphoid lesion scores in comparison to their unvaccinated-challenged counterparts (UnVac/Ch2a, UnVac/Ch2d, and UnVac/Ch2dMhr) at 21 days post challenge (Table 3).

Macroscopically, a minimal fibrinous perihepatic lesion was observed in pigs from the Vac/Ch2dMhr group (Figure 5c). A severe fibrinous perihepatic lesion was observed in pigs from the UnVac/Ch2dMhr group (Figure 5d). Microscopically, a moderate fibrinous perihepatic lesion, covered with moderate non-organized fibrin, was observed in pigs from the Vac/Ch2dMhr group (Figure 6e). Severe fibrinous perihepatic lesion covered with abundant non-organized fibrin was observed in the pigs from the UnVac/Ch2dMhr group (Figure 6f). Pigs in the vaccinated–challenged groups (Vac/Ch2dMhr) had significantly lower (*p* < 0.05) microscopic fibrinous hepatic lesions compared to unvaccinated-challenged groups (UnVa/Ch2dMhr) at 21 days post challenge (Table 3). Pulmonary, lymphoid, and hepatic lesions were not observed in pigs in the negative control group (UnVac/UnCh).

## 4. Discussion

The present experimental challenge study demonstrated that a novel combined vaccine containing PCV2a/2d, *M. hyopneumoniae*, and *M. hyorhinis* effectively protected pigs against challenge with these pathogens. As reported previously, infection with PCV2a or PCV2d alone does not induce PCVAD in pigs [30]. Accordingly, vaccinated pigs challenged solely with PCV2a or PCV2d did not show improved growth performance compared to unvaccinated controls. In contrast, co-infection with PCV2 and *M. hyorhinis* induces full manifestations of PCVAD [18]. Consistently, vaccinated pigs challenged with PCV2d and *M. hyorhinis* exhibited significantly better growth performance than their unvaccinated counterparts. Furthermore, since *M. hyopneumoniae* is known to cause chronic respiratory disease with growth retardation [31], vaccinated pigs challenged with *M. hyopneumoniae* also showed significant improvement in growth performance compared to unvaccinated pigs.

The combined vaccine offers effective protection againstPCV2d, the currently most clinically relevant PCV2 genotype. Although traditional PCV2a-based vaccines have provided acceptable cross-protection against PCV2d in many herds [21], their efficacy has become increasingly unreliable [9,32,33]. Notably, PCVAD outbreaks caused by PCV2d has been reported in herds vaccinated with PCV2a-based products [32,33,34], raising concerns regarding the protective capacity of such vaccines. Accordingly, a combined vaccine containing both PCV2a and PCV2d is expected to provide broader genotypic coverage and superior protection compared to monovalent vaccines under diverse field conditions.

The clinical manifestation of PCVAD is closely associated with viral load in growing pigs. A high PCV2 viremia leads to overt clinical disease with increased mortality, whereas low viremia results in subclinical infection and impaired growth performance [35,36]. Thus, reduction in PCV2 viremia is considered the primary indicator of vaccine efficacy. Control of PCV2a and PCV2d viremia reflect the protective immunity elicited by vaccination, which involves both humoral (i.e., neutralizing antibodies) and cell-mediated (i.e., IFN-γ-SC) responses [37,38,39,40]. In this study, the combined vaccine containing PCV2a/2d antigens induced protective immune responses, significantly reduced PCV2a and PCV2d viremia, and decreased the severity of lymphoid lesions.

In the PCV2d and *M. hyorhinis* co-infection model, the reduction in PCV2d viremia in vaccinated pigs was likely mediated by a direct antiviral immune response involving neutralizing antibodies and cell-mediated immunity. Because Mycoplasma co-infection enhances inflammation, promotes PCV2 replication, and broadens tissue distribution [18], the observed reduction in *M. hyorhinis* load may have indirectly contributed to lower PCV2 replication by mitigating local inflammation. Collectively, these results suggest that the combined vaccine exerts both direct antiviral effects against PCV2 and indirect benefits through suppression of *M. hyorhinis*-induced inflammation, thereby reducing overall viral burden and disease severity.

Vaccination induces a cellular immune response to *M. hyopneumoniae*, which plays a critical role in controlling infection [41]. Therefore, vaccination of pigs with a combined vaccine resulted in the induction of cellular immune response to *M. hyopneumoniae* and demonstrated protection by significantly lowering mycoplasmal laryngeal loads while reducing lung lesions.

In recent years, *M. hyorhinis* has been increasingly recognized as a cause of polyserositis and arthritis, contributing to elevated morbidity and mortality in pigs. Control measures, such as improved management and antimicrobial therapy, can reduce infection pressure but are not always sufficient [5]. Although detailed information on vaccine-mediated protective mechanisms against *M. hyorhinis* remains limited, the combined vaccine evaluated here elicited systemic humoral and cellular responses, reduced nasal *M. hyorhinis* loads, and lessened the severity of polyserositis. Thus, this combined vaccine may represent a valuable alternative tool to controlling *M. hyorhinis* infections in swine herds.

Only four different vaccinated-challenged groups (Vac/Ch2a, VacCh2d, Vac/Ch2dMhr, and Vac/ChMhp) were included in this study, in accordance with recommendations from the Seoul National University Institutional Animal Care and Use Committee, and the guideline of the Republic of Korea’s Animal, Plant & Fisheries Quarantine & Inspection Agency, to minimize animal use. A notable discrepancy exists between PCV2 genotypes circulating globally and those represented in commercial vaccines, raising concerns about inadequate protection, particularly against PCV2d. In the present study, a combined vaccine containing PCV2a/2d, *M. hyopneumoniae*, and *M. hyorhinis* conferred protection in challenge models against all four pathogens. Therefore, this combined vaccine may represent an effective strategy to enhance growth performance and control complex co-infection in swine populations.

## 5. Conclusions

This controlled experimental challenge study demonstrated that a novel combined vaccine comprising PCV2a/2d, *Mycoplasma hyopneumoniae*, and *M. hyorhinis* effectively protected pigs against these pathogens. Vaccination significantly reduced PCV2 viremia, laryngeal and nasal mycoplasma loads, and the severity of associated lesions, while enhancing systemic immune responses and growth performance. Collectively, these findings support the combined vaccine as promising strategy for the integrated control of PCV2-associated diseases, enzootic pneumonia, and *M. hyorhinis*-related conditions, with the potential to improve overall herd health and productivity.

## Figures and Tables

**Table 1:** Experiment design with vaccination protocol and challenge models of porcine circovirus type 2a/d (PCV2a/d), *Mycoplasma hyopneumoniae* (Mhp), and *M. hyorhinis* (Mhr). Abbreviations: IM, intramuscular; IN, intranasal; IT, intratracheal; IP, intraperitoneal; PBS, phosphate-buffered-saline. “−” indicates no challenge of pathogen.

Groups	Vaccination (Days of Age)	Challenge (Days of Age)			
		PCV2a	PCV2d	Mhp	Mhr
Vac/Ch2a	2.0 mL IM vaccination (21)	3.0 mL IN (42)	−	−	−
Vac/Ch2d	2.0 mL IM vaccination (21)	−	3.0 mL IN (42)	−	−
Vac/ChMhp	2.0 mL IM vaccination (21)	−	−	7.0 mL IT (42)	−
Vac/Ch2dMhr	2.0 mL IM vaccination (21)	−	3.0 mL IN (42)	−	2.0 mL IT and IP (42)
UnVac/Ch2a	2.0 mL IM PBS injection (21)	3.0 mL IN (42)	−	−	−
UnVac/Ch2d	2.0 mL IM PBS injection (21)	−	3.0 mL IN (42)	−	−
UnVac/ChMhp	2.0 mL IM PBS injection (21)	−	−	7.0 mL IT (42)	−
UnVac/Ch2dMhr	2.0 mL IM PBS injection (21)	−	3.0 mL IN (42)	−	2.0 mL IT and IP (42)
UnVac/UnCh	2.0 mL IM PBS injection (21)	−	−	−	−

**Table 2:** Average daily weight gain (ADWG, gram/pig/day, mean ± standard deviation) of pigs in different groups. Comparisons were made among three groups within each challenge: Vac/Ch2a, UnVac/Ch2a, and UnVac/UnCh (PCV2a); Vac/Ch2d, UnVac/Ch2d, and UnVac/UnCh (PCV2d); Vac/ChMhp, UnVac/ChMhp, and UnVac/UnCh (*M. hyopneumoniae*); Vac/Ch2dMhr, UnVac/Ch2dMhr, and UnVac/UnCh (PCV2d and *M. hyorhinis*). ^a,b,c^ indicate significant differences (*p* < 0.05) within the same challenge and the same period (group 1 = Vac/Cha2a, UnVac/Ch2a/UnVac/UnCh; group 2 = Vac/Ch2d, UnVac/Ch2d, UnVac/UnCh; group 3 = Vac/ChMhp, UnVac/ChMhp. UnVac/UnCh; group 4 = Vac/Ch2dMhr, UnVac/2dMhr, UnVac/UnCh).

Groups	Average Daily Weight Gain (Days, Unit = gram/pig/day)		
	21–42	42–63	21–63
Vac/Ch2a	166.67 ± 28.57	534.29 ± 43.17	350.48 ± 9.43
UnVac/Ch2a	163.81 ± 15.53	486.67 ± 134.87	325.24 ± 81.05
UnVac/UnCh	166.67 ± 16.77	577.14 ± 71.99	371.9 ± 37.74
Vac/Ch2d	173.33 ± 15.28	520.95 ± 40.77	347.14 ± 21.26
UnVac/Ch2d	162.86 ± 38.73	481.9 ± 147.52	322.38 ± 72.93
UnVac/UnCh	166.67 ± 16.77	577.14 ± 71.99	371.9 ±37.74
Vac/ChMhp	161.9 ± 42.72	524.76± 30.38 ^ab^	343.33 ± 21.26 ^ab^
UnVac/ChMhp	165.71 ± 41.34	380 ± 87.88 ^b^	272.86 ± 40.46 ^b^
UnVac/UnCh	166.67 ± 16.77	577.14 ± 71.99 ^a^	371.9 ± 37.74 ^a^
Vac/Ch2dMhr	160.95 ± 33.23	269.52 ± 25.11 ^b^	215.24 ± 7.82 ^b^
UnVac/Ch2dMhr	158.1 ± 32.49	149.52 ± 60.88 ^c^	153.81 ± 24.66 ^c^
UnVac/UnCh	166.67 ± 16.77	577.14 ± 71.99 ^a^	371.9 ± 37.74 ^a^

**Table 3:** Pathological outcomes (mean ± standard deviation) in different groups at 21 days post challenge. Comparisons were made among three groups within each challenge: Vac/Ch2a, UnVac/Ch2a, and UnVac/UnCh (PCV2a); Vac/Ch2d, UnVac/Ch2d, and UnVac/UnCh (PCV2d); Vac/ChMhp, UnVac/ChMhp, and UnVac/UnCh (*M. hyopneumoniae*); and Vac/Ch2dMhr, UnVac/Ch2dMhr, and UnVac/UnCh (PCV2d and *M. hyorhinis*). ^a,b,c^ indicate significant differences (*p* < 0.05) among groups within the same organ (group 1 = Vac/Cha2a, UnVac/Ch2a, UnVac/UnCh; group 2 = Vac/Ch2d, UnVac/Ch2d, UnVac/UnCh; group 3 = Vac/ChMhp, UnVac/ChMhp. UnVac/UnCh; group 4: Vac/Ch2dMhr, UnVac/Ch2dMhr, UnVac/UnCh).

Groups	Macroscopic Lesion		Microscopic Lesion		
	Lung	Hepatic	Lung	Lymph Node	Hepatic
Vac/Ch2a	NT	NT	NT	2.24 ± 0.65 ^b^	NT
UnVac/Ch2a	NT	NT	NT	3.68 ± 0.95 ^a^	NT
UnVac/UnCh	NT	NT	NT	0 ± 0 ^c^	NT
Vac/Ch2d	NT	NT	NT	2.52 ± 0.83 ^b^	NT
UnVac/Ch2d	NT	NT	NT	3.72 ± 0.63 ^a^	NT
UnVac/UnCh	NT	NT	NT	0 ± 0 ^c^	NT
Vac/ChMhp	13 ± 9.75 ^b^	NT	1.44 ± 0.95 ^b^	NT	NT
UnVac/ChMhp	37 ± 11.51 ^a^	NT	3.76 ± 0.62 ^a^	NT	NT
UnVac/UnCh	0 ± 0 ^b^	NT	0 ± 0 ^c^	NT	NT
Vac/Ch2dMhr	NT	1.6 ± 0.55 ^b^	NT	2.8 ± 0.81 ^b^	4 ± 0.69 ^b^
UnVac/Ch2dMhr	NT	2.8 ± 0.45 ^a^	NT	4.32 ± 0.58 ^a^	4.84 ± 0.26 ^a^
UnVac/UnCh	NT	0 ± 0 ^c^	NT	0 ± 0 ^c^	0 ± 0 ^c^

NT = not tested.

## Data Availability

The data used to support the findings of this study are included within this article.
